# The return of austerity imperils global health

**DOI:** 10.1136/bmjgh-2022-011620

**Published:** 2023-02-20

**Authors:** Thomas Stubbs, Alexandros Kentikelenis, Daniela Gabor, Jayati Ghosh, Martin McKee

**Affiliations:** 1 Department of Politics and International Relations, Royal Holloway, University of London, Egham, UK; 2 Department of Social and Political Sciences, Bocconi University, Milan, Italy; 3 Department of Accounting, Economics and Finance, University of the West of England, Bristol, UK; 4 Department of Economics, University of Massachusetts, Amherst, Massachusetts, USA; 5 Department of Health Services Research and Policy, London School of Hygiene & Tropical Medicine, London, UK

**Keywords:** Health policy, Health systems, Public Health, COVID-19

## Abstract

Recognising the world’s lack of preparedness for the COVID-19 pandemic, international organisations like the World Health Organization, World Bank, and International Monetary Fund are calling for extensive additional funding to strengthen pandemic preparedness and response systems in low-income and middle-income countries, including through domestic resource mobilisation. This article examines the prospects of national health budgets increasing in such a context, drawing on new International Monetary Fund projections on public spending around the world. We show that by 2024 public spending will be lower than the 2010s average for almost half of all low-income and middle-income countries. A key driver of this new wave of austerity is the dramatic increase in public spending dedicated to repaying external debt—underpinned by growing debt stocks, US interest rates rises, and commodity price hikes. As in earlier crises, the stage is set for a situation where population health deteriorates—via compound effects of the pandemic and widespread economic hardship—while public health services required to tackle increased need are facing steep cuts. We conclude by considering what can be done to avoid repeating the mistakes of the past.

**Summary box**

- Transnational organisations are calling for extensive additional funding to strengthen pandemic preparedness and resource systems in low-income and middle-income countries, including through domestic resource mobilisation.
- Economic projections show that austerity is on the agenda for 59 out of 125 low-income and middle-income countries by 2024, diminishing the prospect of national health budgets increasing and threatening pandemic preparedness aspirations.
- A key driver of steep public expenditure reductions is increasing external indebtedness: low-income and middle-income countries will spend more on external debt repayments in 2022 than they spent on health in 2020 during the height of the pandemic.
- Global rules on debt recovery need to change and international financial institutions like the International Monetary Fund and World Bank must enable and support greater public spending to improve population health and achieve universal health coverage.

## Introduction

The world is emerging from the acute phase of the COVID-19 pandemic, a crisis it was wholly unprepared for. As many as 18 million people may have died so far,^1^ with economic losses through 2024 estimated to reach US$13.8 trillion.^2^ The countries with the weakest health and social infrastructure continue to suffer most, measured by damage to health and the economy.^3^

In recent statements, the International Monetary Fund (IMF)—which in effect sets the agenda for global economic policy debates—expressed concern that recovery will be constrained by inadequate support for policies to address the pandemic’s economic and social fallout.^2^ It called for extensive additional efforts and funds—an estimated US$7 billion per year in low-income and middle-income countries (LMICs) for strengthening pandemic preparedness and response systems^4^—to achieve equitable access to healthcare, including through domestic resource mobilisation. This is critical as, before the pandemic, governments were only covering 21% of total health spending in low-income countries and about 35% in middle-income countries.^5^

## What are the prospects of national health budgets increasing?

We sought an answer in the IMF’s October 2022 economic projections for the near term. These are published biannually in the World Economic Outlook, an influential report consulted by policy makers around the world, and especially by close observers of national economies like banks and credit rating agencies, whose decisions determine the ability of governments to raise funds and the terms on which they do so.^6^ In effect, projections become prescriptions for many LMICs, especially those that turn to the IMF for financial support, where they are inscribed as conditions the country must meet for loans to be disbursed. Since January 2021, 46 countries have received such conditional support.

**Figure 1:**
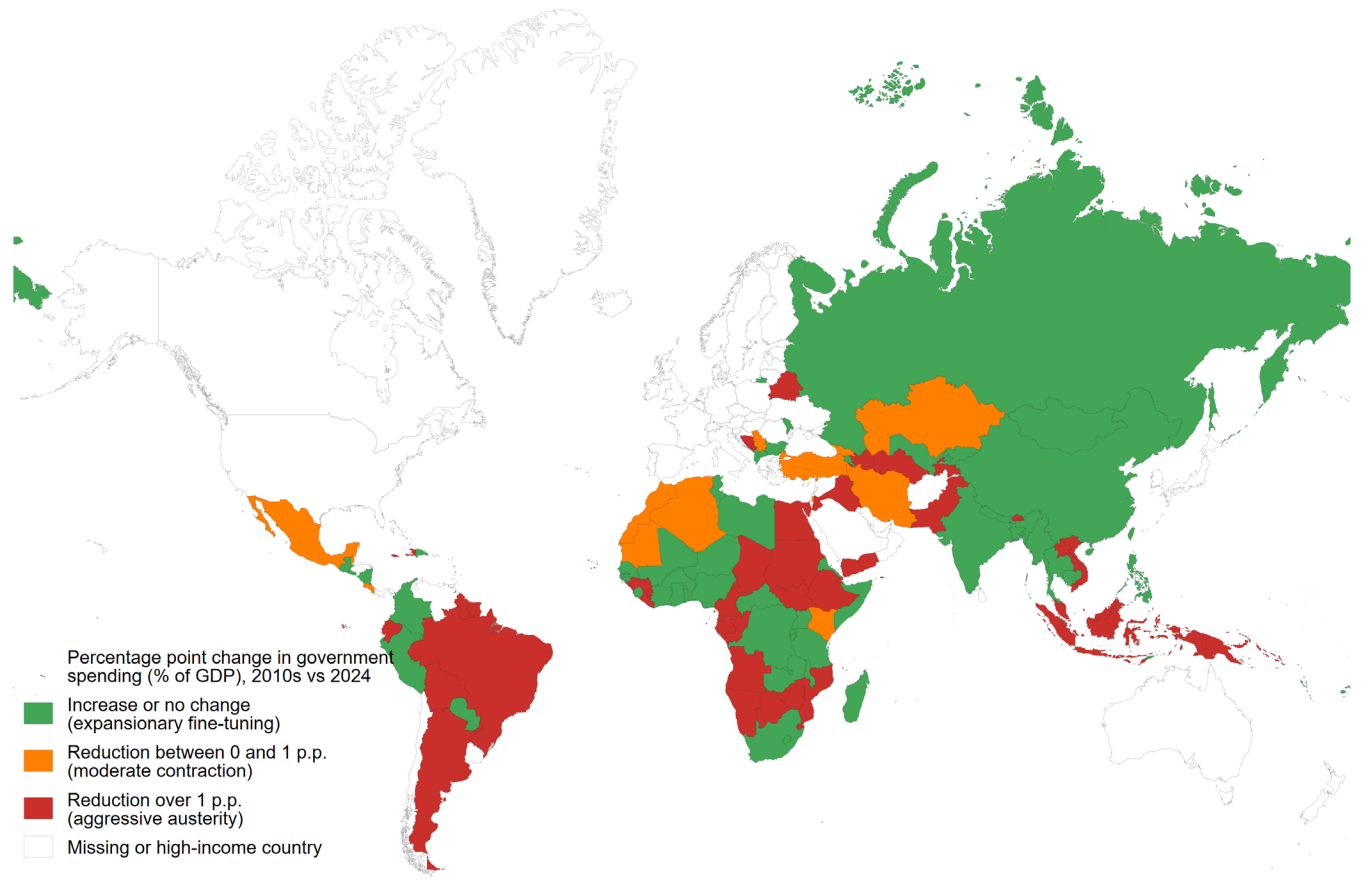
Government expenditure changes as a share of GDP in low-income and middle-income countries, 2010s vs 2024.

The IMF’s public expenditure projections in figure 1 show that austerity is back on the agenda in almost half of LMICs, and this is likely to be an overoptimistic estimate as IMF projections tend to have an optimism bias.^7 8^ Almost all governments had spent more in 2020, averaging a 2.2 percentage point year-on-year increase as a share of GDP to support their citizens and businesses through the acute phase of the pandemic, but this spending is already being rolled back. By 2024, 59 (out of 125) LMICs are expected to spend less than their average expenditure as a share of GDP during the 2010s, exposing a total of 2.0 billion people to the health consequences of budget cuts. Many of those projecting no change or a small increase, as in West and Central Africa, are already spending very little.^9^

This upcoming wave of austerity builds on a legacy of decline in public spending, which accounted for an average 28.6% of GDP in LMICs in 2019, representing a drop of 0.6 percentage points compared with 2010. As a result, public spending on health had, between 2015 and 2019, stagnated: the median LMIC government spent only 2.1% of GDP on health; and the average LMIC resident lived in a country whose government spent 2.0% of GDP on health (calculated on a population-weighted basis).^5^ In other words, the projected cuts will compound the effects of past austerity that, in many cases, had reduced preparedness for the pandemic and may have necessitated even higher countercyclical fiscal efforts when it occurred.

## Which countries will be affected the most?

Individual countries face different challenges—such as conflict or extreme weather events—which in turn affect their risk of being forced into introducing austerity measures. Such an approach is often linked to the IMF’s own prescriptions in the context of its lending programmes. This is the case for approximately one-third of fiscally contracting countries (with more expected to enter IMF programmes in the coming year). But regardless of IMF lending status, a key driver of steep public expenditure reductions is increasing external indebtedness: the World Bank estimates that external service payments on public debt will jump by 35% between 2021 and 2022.^10^ There are three main reasons behind this major increase in sovereign indebtedness.

First, following the 2007–2008 Global Financial Crisis, many LMICs took advantage of low interest rates by taking on new loans to fund major infrastructural projects, advance their development goals, and address the needs of growing populations. As a result, external debt stocks of LMICs increased from US$4.2 to US$8.5 trillion between 2010 and 2020. Debt owed to private creditors is a key driver of this increase, and these creditors have traditionally opposed any changes to the terms of debt service.^10^ In addition, many LMICs issued sovereign bonds, many of which are maturing in the 2022–2024 period.^11^ Then, when COVID-19 hit, LMICs took on additional external debt to finance measures to mitigate its economic and social impact—an average 5.6% increase in nominal terms in 2021, or US$582 billion in additional debt.^10^

Second, interest rate rises in the USA have had knock-on effects on the cost of debt service for LMICs: loan terms are commonly linked to the US interest rate, and the appreciation of the US dollar against LMIC currencies raises the costs of debt denominated in it. This reflects an entire international financial architecture that favours creditors and punishes borrowers. This imbalance is most conspicuous in the case of Global North private sector creditors to developing countries: they account for approximately 61% of the total external debt stock of LMICs and benefited from high risk premia on such debt,^10^ yet they are vehemently—and successfully—opposed to any modicum of debt restructuring that would affect the return on their investments.

Third, there were steep commodity price hikes consequent on the war in Ukraine, thereby diminishing foreign exchange reserves of LMIC importers. After a 16% drop in commodity prices in the first year of the pandemic, 2021 saw a 55% increase, with an estimated further increase of about 56% in 2022, driven by the price of fuel, followed by food.^12^

**Figure 2:**
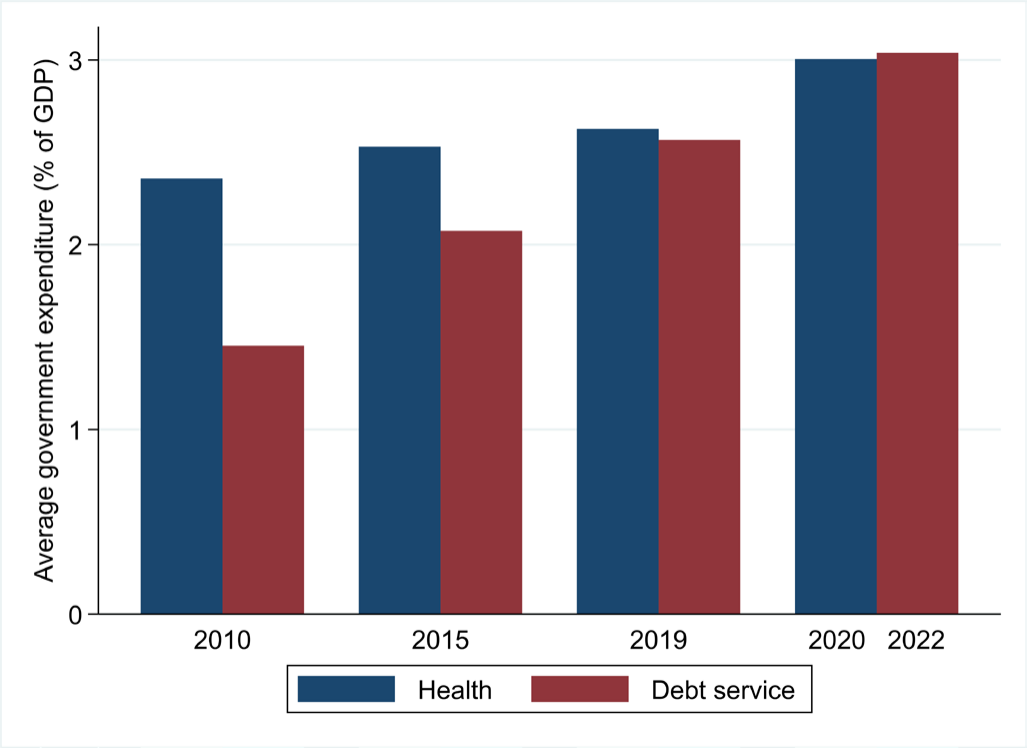
Government expenditure on health and external debt service as a share of GDP in low-income and middle-income countries. Debt service includes public and publicly guaranteed external debt but excludes International Monetary Fund debt service (ie, charges and repurchases) and domestic debt.

As a result of these forces, at least 45 LMICs are considered in or close to debt distress.^12^ As figure 2 shows, LMICs will spend an average 3.04% of GDP on external debt service in 2022,^10^ thereby diverting resources away from investment in public health—higher than the 3.01% that LMICs spent on health in 2020 during the height of the pandemic (the latest year for which data are available), which itself embodied a significant 0.38 percentage point hike from 2019.^5^ This represents a substantial change in the composition of public finances from a decade ago. In 2010, LMIC expenditure on external debt service (1.45% of GDP) represented less than two-thirds of spending on health (2.36%). As the decade progressed, government spending on debt service climbed to become comparable with health spending by 2019; and, for 2022, is double what it was in 2010 as a share of GDP.

Of course, measuring public expenditure as a share of GDP is highly sensitive to changes in GDP and may thus offer a misleading image on the extent of austerity. Converting public spending into a per capita measure presents a near-identical trend. In 2010, LMICs dedicated an average of US$63 per capita to external debt service and US$99 to government health spending. By 2022, US$138 was spent on external debt service compared with US$141 on health in 2020, the peak year for public health spending. To put this spending in perspective, the average high-income country spent US$2361 per capita on health in 2020.

## What are the prospects for improving health and achieving universal health coverage?

The new wave of austerity threatens to undermine progress on improving health in many LMICs. As in earlier major crises, the stage is set for a situation where population health deteriorates—through compound effects of the pandemic and widespread economic hardship—even as the public health services required to tackle increased need face steep cuts. Despite more than a decade of policy attention to ‘health systems strengthening’ as well as lip service by domestic and international policy makers to the importance of health systems since the emergence of COVID-19, there is now a real and imminent danger that these systems will be seriously weakened in almost half of all LMICs.

If this is the case, individuals will likely be faced with a choice between greater out-of-pocket payments for private health services or no medical attention at all. There is some evidence that the latter is happening already. In the first year of the pandemic, out-of-pocket spending in LMICs dropped by about 2%,^5^ and it is unlikely to tick up further given the ongoing cost of living crisis. This means that people who are unable to afford private health services or access public health facilities due to reduced services and longer waiting times are likely to postpone necessary screening or treatments. This will disproportionately affect the poorest and most vulnerable populations,^5^ and will likely exacerbate health inequalities within countries.

But a different way is possible. The global community has committed to universal health coverage by signing up to the United Nation’s Sustainable Development Goals. The COVID-19 pandemic opened a window of opportunity to do things differently, and many LMICs already jettisoned old orthodoxies and embraced more interventionist approaches to protect health. The return of austerity will endanger progress already achieved, as approximately half of LMICs face the prospect of steep cuts.

What can be done? First and most pressingly, governments should reject policies that risk a downward spiral with economic decline and worsening health and education mutually reinforcing each other. This means investing to break this cycle. The role of international financial institutions like the IMF and World Bank is key: they can provide grants and low-interest loans to countries in need and can help legitimate expanded public spending to avoid socioeconomic calamity and invest in the social cohesion and human capital necessary for growth, rather than portraying it as evidence of profligate public officials. The first glimmers of hope for such a revamped approach were set in the early pandemic period: IMF Managing Director Kristalina Georgieva called for countries to spend as much as they can to protect health,^13^ and World Bank Chief Economist Carmen Reinhart advocated that LMICs should ‘first fight the war, then figure out how to pay for it.’^11^ This has coincided with emerging evidence challenging previous beliefs that high debt levels depress economic growth.^14^ The need for such expansionary policies remains.

Second, there is an urgent need to change global rules on debt recovery. This is most pressingly the case for debt owed to the private sector, which has proven highly resistant to rescheduling, renegotiation, or relief attempts. Even though countries in the Global North displayed an initial willingness to reschedule LMIC debts and offered moderate support for debt relief, private financial entities in these countries have steadfastly refused to engage. This has led to a large growth of debt distress: latest available data show that the interest rates on sovereign bonds issued by 19 developing countries are at least 10 percentage points higher than those for US Treasury bonds, up from just three countries in early 2019; this severely restricts their access to international capital markets.^15^ A particular concern has been the emergence of ‘vulture funds’ that buy distressed debt at a large discount and seek to recover it from debtor governments through the courts. While progress has been made in limiting their ability to litigate to recover funds, there is more that could be done.^16^ Relying only on voluntary participation by private creditors is likely to be ineffective, as it has proved to be in the past two years. Without comprehensive debt relief and restructuring of both public and private debt, coupled with grants, concessional financing, and policy reforms, many LMICs will not be able to access and mobilise the necessary resources to support even their current, low levels of health spending.

Finally, sources of financing for health need to be expanded. In the immediate aftermath of the pandemic, 58% of health spending in low-income countries and 28% in middle-income countries hailed from external sources. But while donors have a continued role to play, they cannot be relied on for sustained financing, especially in the context of global economic turmoil and the politicisation of aid flows in the Global North.^17^ For this reason, domestic resource mobilisation is now more important than ever. Select developing countries have already begun introducing wealth taxes and are seeking to expand what are often thin tax bases. In Argentina, a one-off wealth tax on the richest residents raised US$2.4 billion, about 0.5% of the country’s GDP, for pandemic recovery,^18^ while strict capital controls prevented assets or money from flowing out of the country.^19^ And Colombia is currently revamping its tax system to generate sustainable sources of finance for health and other social policies.^20^ Such policies provide encouraging models that could be emulated elsewhere. However, they also require concerted action to tackle corruption,^21^ including measures in the Global North, where financial centres facilitate money laundering and capital flight.^22^ In this respect, health policy advocates should engage with the work of investigative journalists such as those who have revealed widespread corruption in, for example, the Panama and Paradise papers.^23^ In addition, the creation of national asset registers that identify the beneficial owners, and the cross-country sharing of such data, are crucial.

Austerity imperils efforts to improve health and endangers progress already achieved. These are not novel insights, but well-documented findings from voluminous public health scholarship that has demonstrated the heavy toll of rapid and radical budget cuts on health policy and population health.^24–27^ Repeating the mistakes of the past is not only misguided but also short-sighted. As UNAIDS Executive Director Winnie Byanyima recently stressed, ‘the risks from future pandemics (are) so grave, that we cannot afford not to protect ourselves.’^28^ Austerity measures exacerbate these risks, and undermine hard-won improvements in health.

10.1136/bmjgh-2022-011620.supp1 Supplementary data

## Data Availability

All data are drawn from public datasets and are available as supplementary material to this article.
